# Supplementary material for: SNP-Based Heritability of Osteochondrosis Dissecans in Hanoverian Warmblood Horses
Source: Animals (Basel). 2023 Apr 25;13(9):1462. doi: 10.3390/ani13091462 (PMC10177438; doi:10.3390/ani13091462)
Supplement: Supplementary file 1 [file animals-13-01462-s001.zip › animals-2283989-supplementary.pdf]

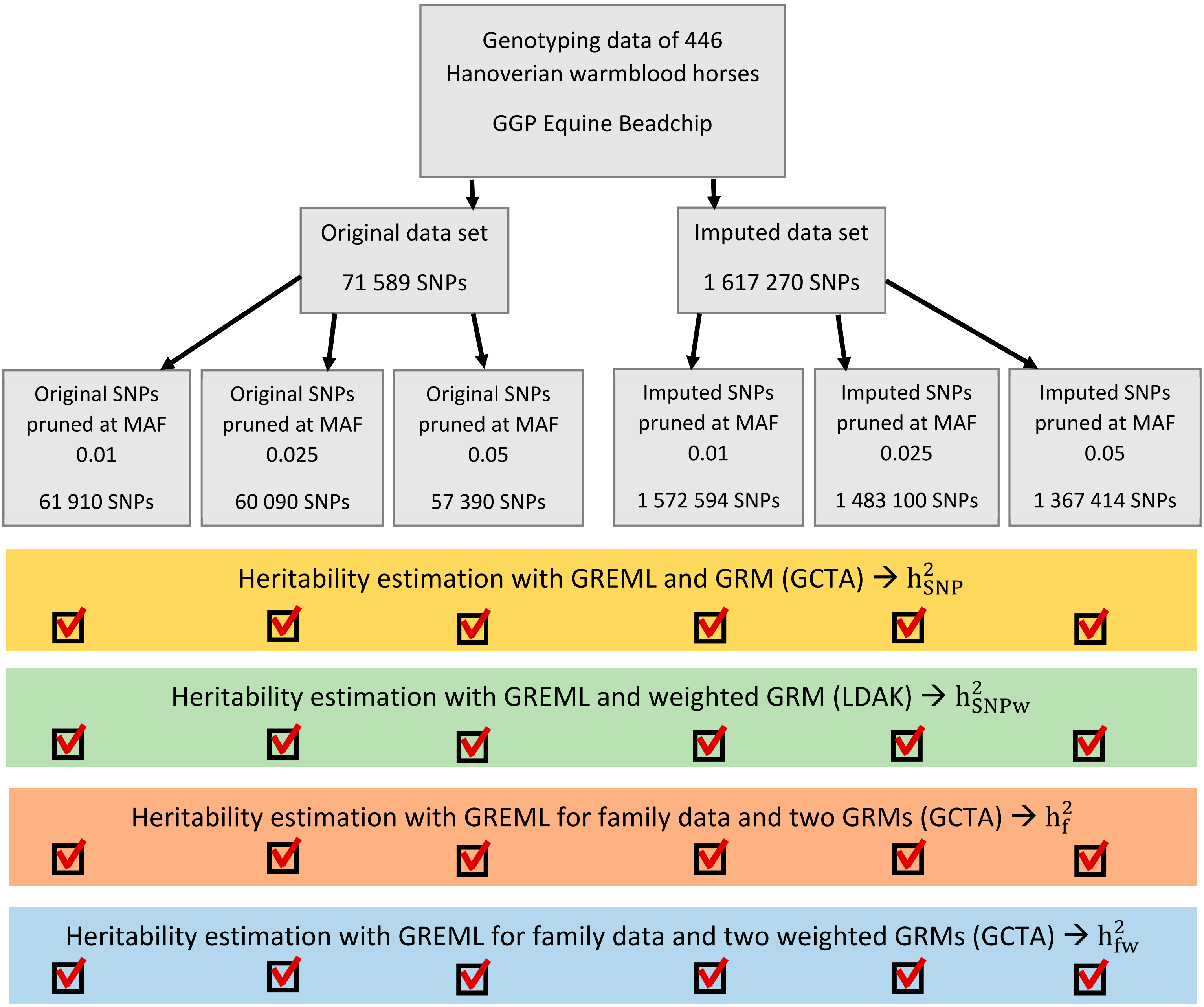

Figure S1. Survey on the study design to estimate heritabilities using different approaches and genotype data.

Table S1. Estimates of heritability for equine osteochondrosis reported in previous studies

Population and number of investigated horses	Radiographic finding	Heritability estimate	Method of analysis	References
**(Australian and New Zealand) Thoroughbred horses (n=1004)**	OC<br>OC fore fetlock<br>OC hock<br>OC stifle	0.11<br>0.15<br>0.10<br>0.10	GLMM	Castle (2012) [21]
**Norwegian Standardbred trotters (n=1217)**	OC DIT/LTT<br>OC DIT	0.29 ± 0.15<br>0.40 ± 0.17	STM, DL	Lykkjen et al. (2013) [20]
**Hanoverian warmblood horses (n=7396)**	OC fetlock<br>OC hock<br>OC stifle<br>OCD fetlock<br>OCD hock<br>OCD stifle	0.167 ± 0.032<br>0.345 ± 0.039<br>0.205 ± 0.043<br>0.162 ± 0.038<br>0.462 ± 0.058<br>0.234 ± 0.070	LAM, DL	Hilla and Distl (2013) [5]
**(Australian) Thoroughbred horses (n=1962)**	OC<br>OC stifle<br>OC hock<br>OC fetlock	0.08<br>0.16<br>0.00<br>0.02	LSM, DL	Russell et al. (2017) [19]
**Polish Warmblood horses (n=201)**	OCD	0.30-0.27	SNP, LAM, DL	Lewczuk et al. (2017) [22]
**North American Standardbred horses (n=479)**	OC hock	0.24 ± 0.16	SNP, GREML	McCoy et al. (2019) [23]

DIT, distal intermediate ridge of the tibia; LTT, lateral trochlear ridge of the talus; GLMM, generalised linear mixed model; STM, Sire threshold model; REML, Restricted Maximum Likelihood; LSM, Linear sire model; DL, Dempster Lerner transformation onto the liability model; LAM, Linear animal model; SNP, SNP-based genome-wide genotyping; GREML, genomic restricted maximum likelihood

Table S2. Publicly available whole genome sequencing data of horses used for imputation.

Bioproject	Biosample	Breed
PRJNA230019	SAMN02422919	Dülmen Horse
PRJNA230019	SAMN02439777	Arabian
PRJNA230019	SAMN02439778	Sorraja
PRJNA230019	SAMN02439779	Hanoverian
PRJNA230019	SAMN02439779	Hanoverian
PRJNA291776	SAMN03952747	Hanoverian
PRJNA291776	SAMN03952758	Hanoverian
PRJNA291776	SAMN03955412	Heavy Warmblood
PRJNA205517	SAMN02179860	Arabian
PRJNA277030	SAMN02179856	Fjord
PRJNA277030	SAMN02179441	Standardbred
PRJNA277030	SAMN02179857	Icelandic
PRJNA246445	SAMN02767683	Marwari
PRJNA273402	SAMN03291442	Connemara
PRJNA273402	SAMN03291461	Connemara
PRJNA430351	SAMN08116965	Hanoverian
PRJNA430351	SAMN08116967	Hanoverian
PRJNA430351	SAMN08116969	English Thoroughbred
PRJNA430351	SAMN08116970	Hanoverian
PRJNA430351	SAMN08116971	Hanoverian
PRJNA430351	SAMN08116972	Hanoverian
PRJNA430351	SAMN08116987	Hanoverian
PRJNA430351	SAMN08116973	Hanoverian
PRJNA430351	SAMN08116989	Hanoverian
PRJNA430351	SAMN08116988	Hanoverian
PRJNA430351	SAMN08116991	Oldenburg
PRJNA430351	SAMN08116990	Hanoverian
PRJNA430351	SAMN08116993	Hanoverian
PRJNA430351	SAMN08116992	Hanoverian
PRJNA430351	SAMN08116995	Oldenburg

Table S3. Estimates for $h^2_{SNP-all-pairs}$ and $h^2_{SNP-all-pairs-w}$ with their standard errors for osteochondrosis dissecans in the fetlock joint estimated with GCTA GREML for family data and GCTA GREML for family data with a LD-weighted genomic relationship matrix with the original and imputed SNP data and at minor allele frequencies of 0.01, 0.025 and 0.05, including transformation to the liability scale (obs = observed scale, liab = liability scale).

Approach	Data set	MAF 0.01		MAF 0.025		MAF 0.05	
		Obs	Liab	Obs	Liab	Obs	Liab
GREML fam ($h^2_{SNP-all-pairs} \pm$ SE)	original	0.16 ± 0.18	0.30 ± 0.34	0.15 ± 0.18	0.28 ± 0.33	0.09 ± 0.17	0.18 ± 0.32
	imputed	0.12 ± 0.16	0.23 ± 0.30	0.10 ± 0.16	0.19 ± 0.29	0.09 ± 0.15	0.17 ± 0.28
GREML fam LD-weighted ($h^2_{SNP-all-pairs-w} \pm$ SE)	original	0.16 ± 0.18	0.29 ± 0.34	0.14 ± 0.18	0.27 ± 0.33	0.14 ± 0.18	0.26 ± 0.33
	imputed	0.12 ± 0.16	0.23 ± 0.30	0.10 ± 0.16	0.19 ± 0.29	0.09 ± 0.15	0.17 ± 0.28

Table S4. Estimates for $h^2_{SNP-all-pairs}$ and $h^2_{SNP-all-pairs-w}$ with their standard errors for osteochondrosis dissecans in the hock joint estimated with GCTA GREML for family data and GCTA GREML for family data with a LD-weighted genomic relationship matrix with the original and imputed SNP data and at minor allele frequencies of 0.01, 0.025 and 0.05, including transformation to the liability scale (obs = observed scale, liab = liability scale).

Approach	Data set	MAF 0.01		MAF 0.025		MAF 0.05	
		Obs	Liab	Obs	Liab	Obs	Liab
GREML fam ($h^2_{SNP-all-pairs} \pm$ SE)	original	0.53 ± 0.18	0.91 ± 0.31	0.49 ± 0.18	0.84 ± 0.30	0.44 ± 0.17	0.76 ± 0.30
	imputed	0.49 ± 0.16	0.84 ± 0.28	0.46 ± 0.16	0.79 ± 0.27	0.44 ± 0.15	0.75 ± 0.25
GREML fam LD-weighted ($h^2_{SNP-all-pairs-w} \pm$ SE)	original	0.50 ± 0.18	0.86 ± 0.31	0.49 ± 0.18	0.83 ± 0.30	0.49 ± 0.18	0.83 ± 0.30
	imputed	0.57 ± 0.18	0.98 ± 0.31	0.58 ± 0.18	1.00 ± 0.31	0.54 ± 0.18	0.92 ± 0.30

Table S5. Estimates for for $h^2_{SNP-all-pairs}$ and $h^2_{SNP-all-pairs-w}$ with their standard errors for osteochondrosis dissecans in the stifle joint estimated with GCTA GREML for family data and GCTA GREML for family data with a LD-weighted genomic relationship matrix with the original and imputed SNP data and at minor allele frequencies of 0.01, 0.025 and 0.05, including transformation to the liability scale (obs = observed scale, liab = liability scale).

Approach	Data set	MAF 0.01		MAF 0.025		MAF 0.05	
		Obs	Liab	Obs	Liab	Obs	Liab
GREML fam ($h^2_{SNP-all-pairs} \pm$ SE)	original	0.20 ± 0.19	1.31 ± 1.23	0.24 ± 0.19	1.53 ± 1.24	0.22 ± 0.19	1.42 ± 1.20
	imputed	0.02 ± 0.16	0.15 ± 1.00	0.02 ± 0.15	0.15 ± 0.96	<0.001 ± 0.15	<0.001 ± 0.93
GREML fam LD-weighted ($h^2_{SNP-all-pairs-w} \pm$ SE)	original	0.14 ± 0.19	0.91 ± 1.19	0.13 ± 0.18	0.86 ± 1.17	0.14 ± 0.18	0.90 ± 1.17
	imputed	0.13 ± 0.19	0.84 ± 1.20	0.07 ± 0.17	0.47 ± 1.12	0.07 ± 0.17	0.49 ± 1.12
